# Immediate protein expression from exogenous mRNAs in embryonic brain

**DOI:** 10.1038/s41598-022-21668-5

**Published:** 2022-10-13

**Authors:** Masae Naruse, Tetsuichiro Saito

**Affiliations:** Department of Developmental Biology, Graduate School of Medicine, Chiba University, Chiba, 260-8670 Japan

**Keywords:** Transfection, Transfection, RNA vaccines

## Abstract

mRNA vaccines for SARS-CoV-2 have been widely used and saving millions of people in the world. How efficiently proteins are produced from exogenous mRNAs in the embryonic brain, however, is less known. Here we show that protein expression occurs highly efficiently in neural stem cells, in a very narrow time window after mRNA electroporation in the embryonic mouse brain, where plasmids have been successfully transfected. Protein expression is detected 1 h and 12 h after the electroporation of mRNAs and plasmids, respectively. The delivery of exogenous mRNAs may be useful for not only vaccines but also functional analysis in the brain.

## Introduction

In vitro synthesized mRNAs that contain modified nucleotides and are encapsulated by lipid nanoparticles (LNPs) have been quickly developed as effective vaccines for SARS-CoV-2^1,2^ and globally deployed^3^. The incorporation of modified nucleotides such as N1-methylpseudouridine (m1ψ) and 5-methoxyuridine (5moU) in place of uridines of mRNAs has been shown to reduce their immunoreactivity and enhance protein expression in vivo^4–7^. LNPs protect mRNAs from degradation and facilitate their delivery into cells, thereby enhancing expression^8^. Protein expression persists for several days after LNP-encapsulated mRNAs are injected into mice^9^, possibly because the mRNAs are stabilized in the LNPs. The injection of an LNP-encapsulated mRNA into the adult mouse ventricle leads to protein expression in neurons^10^. However, how long naked exogenous mRNAs are maintained in embryonic brain cells and how efficiently proteins are translated from the mRNAs in the embryonic brain remain elusive. Embryonic mouse electroporation has been widely used to transfect plasmids into the developing brain.^11^ In this paper, we have examined protein expression from naked exogenous mRNAs by transfecting them into the embryonic mouse brain, using in utero electroporation.

## Results

**Figure 1:**
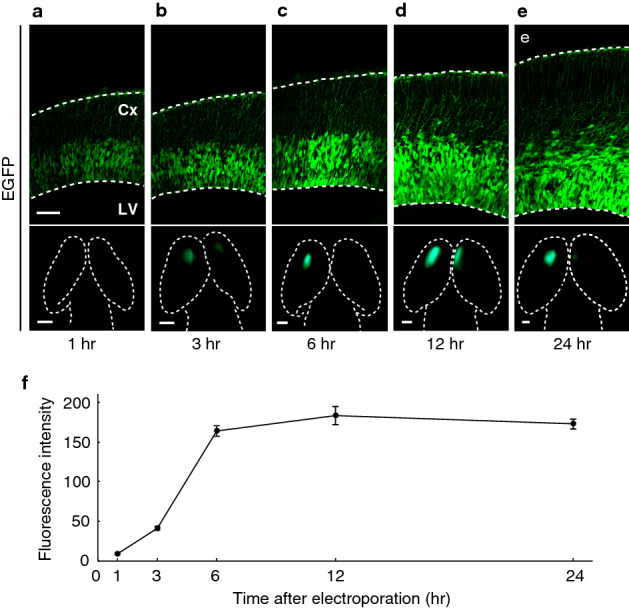
Immediate EGFP expression after mRNA electroporation. The *EGFP* mRNA containing 5moU was injected into the lateral ventricle (LV) of the E13.5 mouse brain, followed by electroporation. (**a**–**e**) Coronal sections of the cerebral cortex (Cx) (upper panel) and dorsal views of the brain (lower panel), one to 24 h after electroporation. Many EGFP-positive cells were observed in the ventricular zone. The EGFP fluorescence 1 h after electroporation was too feeble to detect under the fluorescent stereomicroscope. Scale bars: 50 µm (upper panel), 500 µm (lower panel). (**f**) Fluorescence intensity of EGFP-positive areas, one to 24 h after electroporation. Data are presented as mean ± s.e.m. *n* = 5 electroporated brains.

To examine whether mRNA electroporation is effective for protein expression in the brain, the mRNA of the enhanced green fluorescent protein (EGFP) containing 5moU was injected into the lateral ventricle of the embryonic (E)13.5 mouse brain and electroporated. EGFP fluorescence was observed 1 h after electroporation (Fig. 1), and it reached a plateau 6 h after electroporation. By contrast, EGFP fluorescence was detected 12 h but not 6 h after the electroporation of the plasmid pCAG-EGFP, which carries *EGFP* downstream of the ubiquitous CAG promoter, and it increased with time (Supplementary Fig. S1), suggesting that the EGFP, which is relatively stable, accumulates after translation from the mRNA that is transcribed from the plasmid in vivo.

Electroporation of the *mCherry* mRNA containing 5moU showed a similar time course of expression (Supplementary Fig. S2). The mCherry protein was already produced but its fluorescence was not detected 1 h after electroporation, probably reflecting that the mCherry protein matures more slowly than the EGFP^12^. These findings suggest that the electroporation of mRNAs leads to immediate protein expression in vivo.

Compared with the sustained increase of fluorescence intensity after plasmid electroporation (Supplementary Fig. S1), fluorescence intensity obtained by mRNA electroporation peaked earlier (Fig. 1 and Supplementary Fig. S2), suggesting that mRNA electroporation allows for temporally restricted protein expression, probably because mRNAs are more unstable than plasmids.

**Figure 2:**
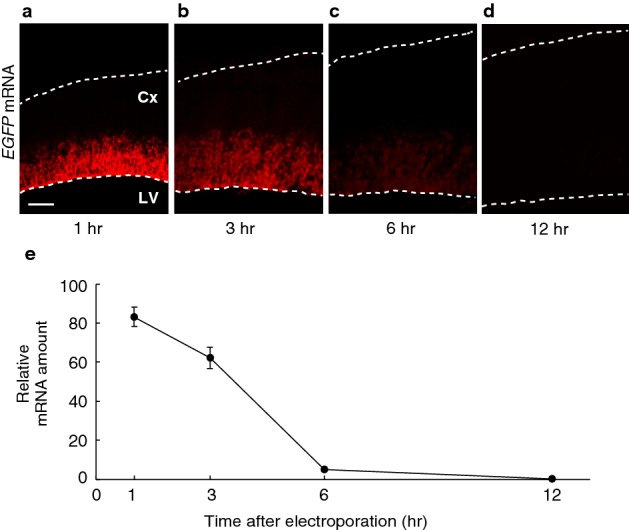
Rapid decay of the exogenous mRNA in cells. (**a**–**d**) FISH images of coronal sections of the Cx, one to 12 h after electroporation, which was performed as described in Fig. 1. The *EGFP* mRNA was detected in the ventricular zone. Scale bar, 50 µm. (**e**) The relative amount of the *EGFP* mRNA, one to 12 h after electroporation, which was measured using fluorescent signals of FISH. Data are presented as mean ± s.e.m. *n* = 5 electroporated brains.

To examine how long the naked exogenous mRNA was maintained in cells, the mRNA was visualized by fluorescence in situ hybridization (FISH). The mRNA rapidly decreased and plateaued 6 h after electroporation (Fig. 2), which was inversely correlated with protein expression (Fig. 1 and Supplementary Fig. S2).

Next, we examined protein expression of the *d2EYFP* mRNA containing m1ψ, which encodes the destabilized enhanced yellow fluorescent protein, half-life of which is approximately 2 h^13^ (Supplementary Fig. S3). d2EYFP fluorescence was detected 1 h after electroporation, increased with time, and started to decrease earlier than those of the *EGFP* and *mCherry* mRNAs, which encode stable proteins.

In the embryonic brain, NSCs undergo cell divisions, in which their nuclei move between the apical and basal sides of the ventricular zone in association with the cell cycle^14^. mRNA electroporation was performed at E13.5, at which stage many neurons are generated, and the cell cycle of NSCs is 11.4 h^15^. One hour after electroporation, NSCs, which were labeled with their marker Sox2, expressed the EGFP in the area adjacent to the ventricle where they divide (Supplementary Fig. S4), suggesting that mRNA electroporation enables NSCs to express exogenous proteins immediately, which was impossible for plasmid electroporation.

## Discussion

This study shows that highly efficient protein expression occurs in a very narrow time window after mRNA electroporation in the embryonic mouse brain. It has been reported that both 5moU and m1ψ increase stability and translation efficiency of mRNA^4–7^. We used mRNAs containing 5moU or m1ψ for electroporation. Immediate protein expression was observed after the electroporation of the both mRNAs containing 5moU and m1ψ, suggesting the narrow time window of protein expression, regardless of whether modified nucleotides are 5moU or m1ψ. The short life and temporally restricted protein expression of exogenous mRNAs in cells may be beneficial for mRNA vaccines as their adverse effects, if any, might be minimized once they are released into cells from LNPs.

In the embryonic mouse brain, NSCs rapidly divide by either symmetric or asymmetric modes^16^. Immediate protein expression by mRNA electroporation may be useful for functional analysis in such rapidly dividing cells.

## Materials and methods

### mRNAs

The *EGFP* and *mCherry* mRNAs containing 5moU were purchased from TriLink Biotechnologies. The *d2EYFP* mRNA was synthesized from the mRNAExpress™ Vector (Systems Biosciences) carrying *d2EYFP* (Clontech), using the MEGAscript™ T7 Transcription Kit (Invitrogen) in the presence of m1ψ (TriLink Biotechnologies), capped with the Vaccinia Capping System and mRNA Cap2-O-Methyltransferase (New England Biolabs), and purified with the MEGAclear Transcription Clean-Up Kit (Thermo Fisher Scientific), according to the manufactures’ protocol.

### Ethical approval

All experimental procedures were approved by the Animal Care and Use Committee at Chiba University (approval number: 4-386) and conducted in accordance with the Guidelines for Use of Laboratory Animals (Japan Neuroscience Society) and the ARRIVE guidelines. All efforts were made to minimize the animals’ suffering. ICR mice (CLEA Japan) were used for all experiments in this study. Mice were housed under standard environmental conditions, maintained on a 12-h light/dark cycle (light on from 6 am to 6 pm) with ad libitum access to food and water. At least 5 electroporated brains from different dams were used for each experiment. A total of twenty-eight pregnant mice were used.

### In utero electroporation and measurement of fluorescence

In utero electroporation was performed as described previously^11^. Time-mated pregnant ICR female mice were deeply anesthetized with a mixture of 40 mg/kg pentobarbital sodium salt (Tokyo Chemical Industry) and 5 mg/kg butorphanol (Vetorphale, Meiji Seika Pharma). One µg/µl of pCAG-EGFP or each mRNA was injected into the lateral ventricle of the E13.5 mouse brain, and five pulses of 40 V were delivered for electroporation. Embryos were dissected 1 h to 24 h after electroporation. Pregnant mice were euthanized by cervical dislocation, and embryos were euthanized by decapitation. Then brains were dissected. Fluorescent images of the dissected brains were captured with the fluorescent stereo microscope M205FA and LAS X software (Leica), then fixed for 1.5 h in 4% paraformaldehyde at 4 °C, submerged in 30% sucrose/PBS and embedded in OCT compound (Sakura, Tokyo, Japan). A cryostat was used to slice 12 μm coronal sections of the cerebral cortex. Fluorescent images of the sections were captured and analyzed with the confocal microscope TCS SP8 and LAS X software (Leica). Mean fluorescence intensity (/µm^2^) of fluorescent positive areas was measured. Mean background fluorescence intensity was obtained by measuring a contralateral untransfected fluorescent-negative area of the same section and subtracted from the mean fluorescence intensity. Each of the fluorescence intensity is an average of the background-subtracted mean fluorescence intensity. The 488 nm and 552 nm lasers were used to excite EGFP and d2EYFP, and mCherry and Fast Red, respectively. Laser powers that did not saturate fluorophores were used. Data are presented as mean ± s.e.m.

### Fluorescence in situ hybridization

In situ hybridization was performed as described previously^17^. In brief, 12 μm sections were treated with 1 μg/ml proteinase K at room temperature for 10 min and hybridized with a digoxygenin (DIG)-labeled *EGFP* probe in the hybridization solution containing 50% formamide, 10 mM Tris–HCl pH 7.5, 600 mM NaCl, 1 mM EDTA, 5% dextran sulfate, 200 μg/ml yeast tRNA, 1 × Denhardt's solution and 0.25% SDS at 65 °C overnight. After washing, the sections were incubated with the alkaline phosphatase-conjugated anti-DIG antibody (#11093274910, Roche) at 4 °C overnight, washed, incubated with SIGMAFAST Fast Red TR/Naphthol AS-MX (Sigma) at room temperature for 1 h, and washed with PBS to remove the substrate.

### Immunohistochemistry

Immunohistochemistry was performed as described previously^17^. In brief, sections on glass slides were treated in 10 mM Tris–HCl pH 9.0, 1 mM EDTA at 95 °C for one minute, incubated with the blocking buffer (10% normal donkey serum, 0.1% Triton-X100 in PBS), incubated with the primary antibodies at 4 °C overnight, washed, incubated with the secondary antibodies at room temperature for 1 h, and washed. The following antibodies were used; mouse anti-mCherry (#632543, Clontech), goat anti-Sox2 (AF2018, R&D Systems), mouse anti-GFP (#11814460001, Sigma), anti-mouse IgG conjugated to Alexa 594 (A21203, Molecular Probe) and anti-mouse IgG conjugated to Alexa 488 (A21202, Molecular Probe), anti-goat IgG conjugated to Alexa 594 (A11058, Molecular Probe). Hoechst 33342 (H1399, Molecular Probe) was used for nuclear staining.

## Supplementary Information

Supplementary Information.

## Data Availability

The datasets used and/or analyzed during the current study and supporting the conclusions of this article are included in this article. These datasets are also available from the corresponding author on reasonable request.

## References

[1] Polack FP (2020). Safety and efficacy of the BNT162b2 mRNA Covid-19 vaccine. N. Engl. J. Med..

[2] Baden LR (2021). Efficacy and safety of the mRNA-1273 SARS-CoV-2 vaccine. N. Engl. J. Med..

[3] Mallapaty S, Callaway E, Kozlov M, Ledford H, Pickrell J, Van Noorden R (2021). How COVID vaccines shaped 2021 in eight powerful charts. Nature.

[4] Pardi N, Hogan MJ, Porter FW, Weissman D (2018). mRNA vaccines - a new era in vaccinology. Nat. Rev. Drug Discov..

[5] Vaidyanathan S (2018). Uridine depletion and chemical modification increase Cas9 mRNA activity and reduce immunogenicity without HPLC purification. Mol. Ther. Nucleic Acids.

[6] Andries O (2015). N(1)-methylpseudouridine-incorporated mRNA outperforms pseudouridine-incorporated mRNA by providing enhanced protein expression and reduced immunogenicity in mammalian cell lines and mice. J. Control Release.

[7] Karikó K (2008). Incorporation of pseudouridine into mRNA yields superior nonimmunogenic vector with increased translational capacity and biological stability. Mol. Ther..

[8] Hou X, Zaks T, Langer R, Dong Y (2021). Lipid nanoparticles for mRNA delivery. Nat. Rev. Mater..

[9] Pardi N (2015). Expression kinetics of nucleoside-modified mRNA delivered in lipid nanoparticles to mice by various routes. J. Control Release.

[10] Lin CY (2016). Messenger RNA-based therapeutics for brain diseases: An animal study for augmenting clearance of beta-amyloid by intracerebral administration of neprilysin mRNA loaded in polyplex nanomicelles. J. Control Release.

[11] Saito T (2006). In vivo electroporation in the embryonic mouse central nervous system. Nat. Protoc..

[12] Macdonald PJ, Chen Y, Mueller JD (2012). Chromophore maturation and fluorescence fluctuation spectroscopy of fluorescent proteins in a cell-free expression system. Anal. Biochem..

[13] Li X (1998). Generation of destabilized green fluorescent protein as a transcription reporter. J. Biol. Chem..

[14] Taverna E, Huttner WB (2010). Neural progenitor nuclei IN motion. Neuron.

[15] Takahashi T, Nowakowski RS, Caviness VS (1995). The cell cycle of the pseudostratified ventricular epithelium of the embryonic murine cerebral wall. J. Neurosci..

[16] Homem CC, Repic M, Knoblich JA (2015). Proliferation control in neural stem and progenitor cells. Nat. Rev. Neurosci..

[17] Naruse M (2006). Induction of oligodendrocyte progenitors in dorsal forebrain by intraventricular microinjection of FGF-2. Dev. Biol..

