# Supplementary material for: Immediate protein expression from exogenous mRNAs in embryonic brain
Source: Sci Rep. 2022 Oct 13;12:17145. doi: 10.1038/s41598-022-21668-5 (PMC9558027; doi:10.1038/s41598-022-21668-5)
Supplement: Supplementary file 1 — Supplementary Information. [file 41598_2022_21668_MOESM1_ESM.pdf]

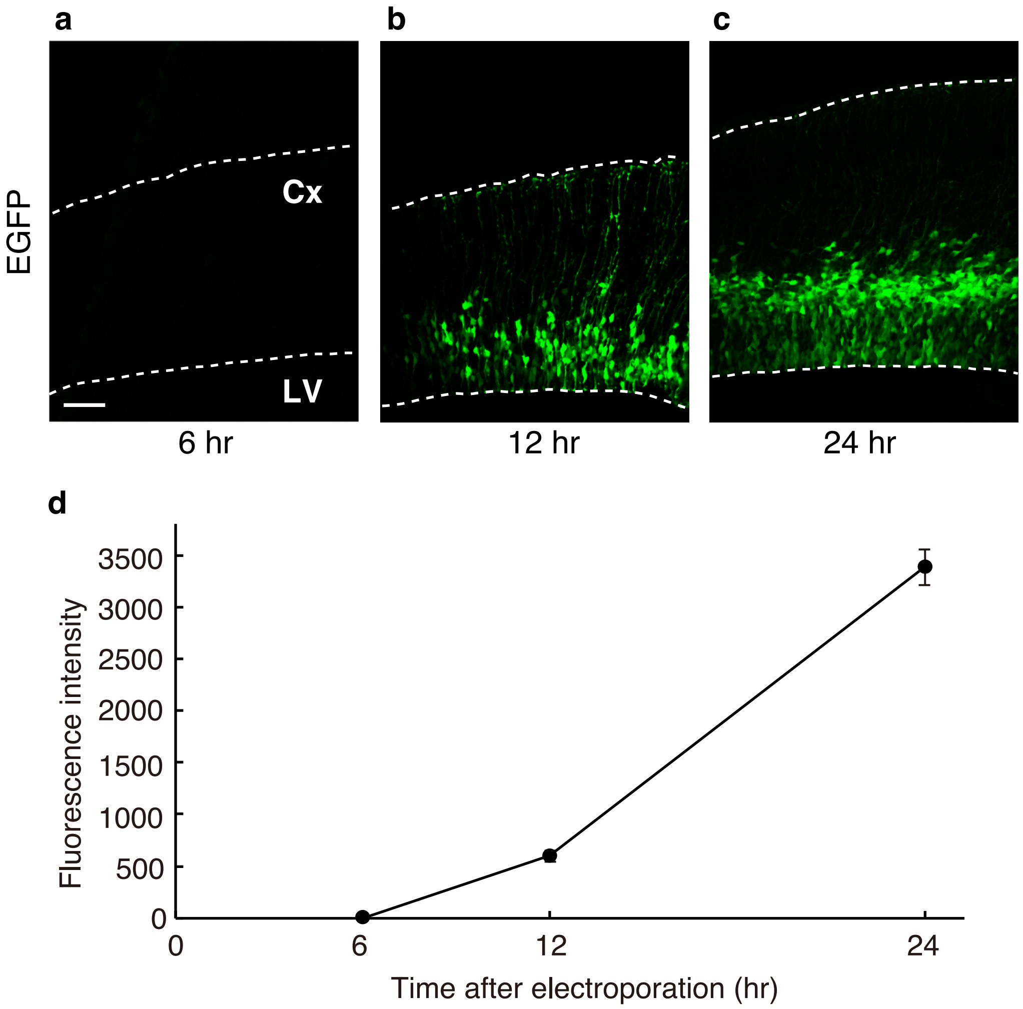

Supplementary Fig. S1. EGFP expression with a lag phase after DNA electroporation. The plasmid pCAG-EGFP was transfected as described in Fig. 1. **a-c,** Coronal sections of the cerebral cortex (Cx), six to 24 hours after electroporation. EGFP fluorescence was not detectable in the sections of the brain six hours after electroporation (*n* = 10 electroporated brains) (**a**). EGFP-positive cells were observed in the ventricular zone 12 hours after electroporation. Scale bar, 50 µm. **d,** Fluorescence intensity of EGFP-positive areas, six to 24 hours after electroporation. Data are presented as mean ± s.e.m. *n* = 5 electroporated brains.

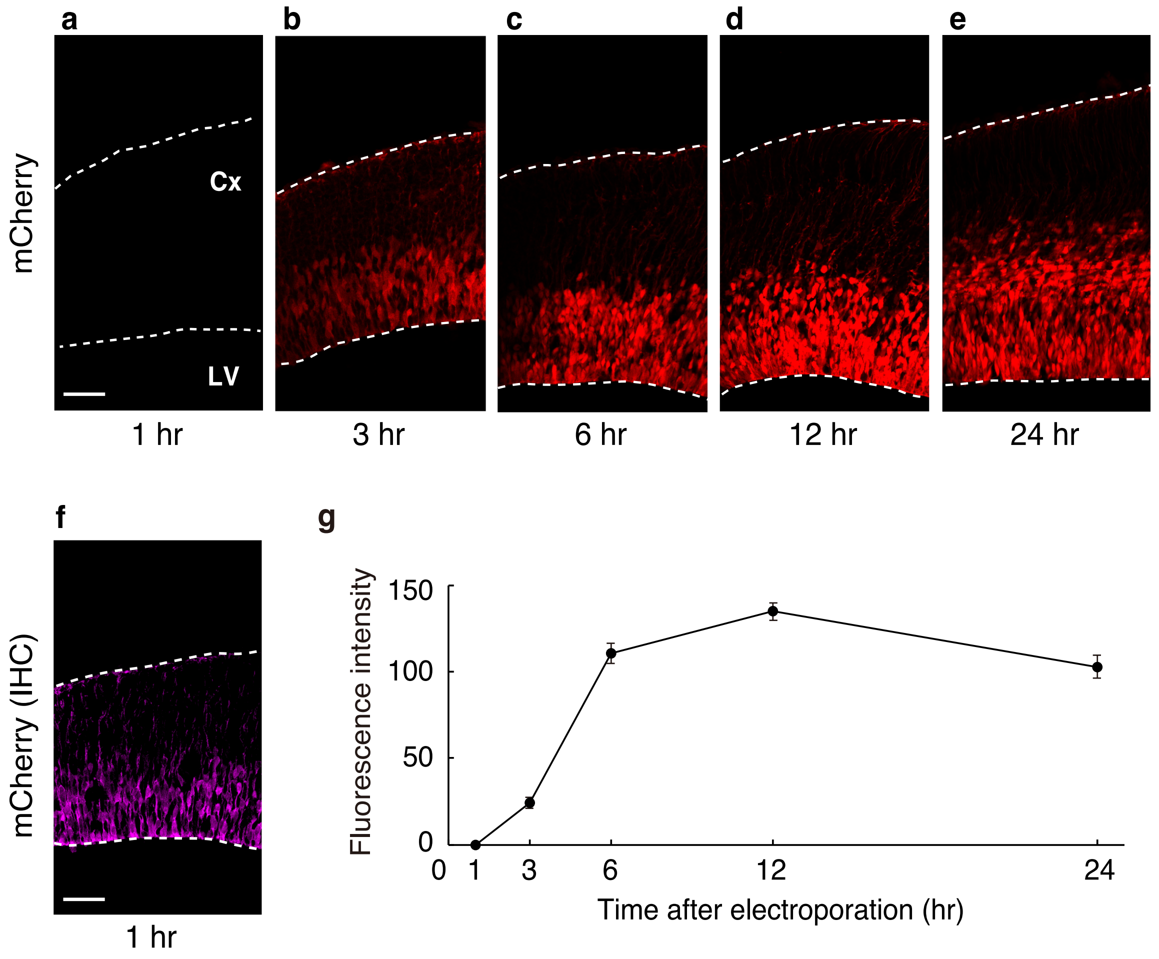

Supplementary Fig. S2. Immediate mCherry expression after mRNA electroporation. The *mCherry* mRNA containing 5moU was transfected as described in Fig. 1. **a-e,** Coronal sections of the Cx, one to 24 hours after electroporation. mCherry fluorescence was not detectable one hour after electroporation (n = 13 electroporated brains) (**a**). Many mCherry-fluorescent cells were observed in the ventricular zone three to 24 hours after electroporation. **f,** Immunohistochemistry (IHC) for the mCherry protein. Scale bars, 50 µm. **g,** Fluorescence intensity of mCherry-positive areas, one to 24 hours after electroporation. Data are presented as mean ± s.e.m. n = 5 electroporated brains.

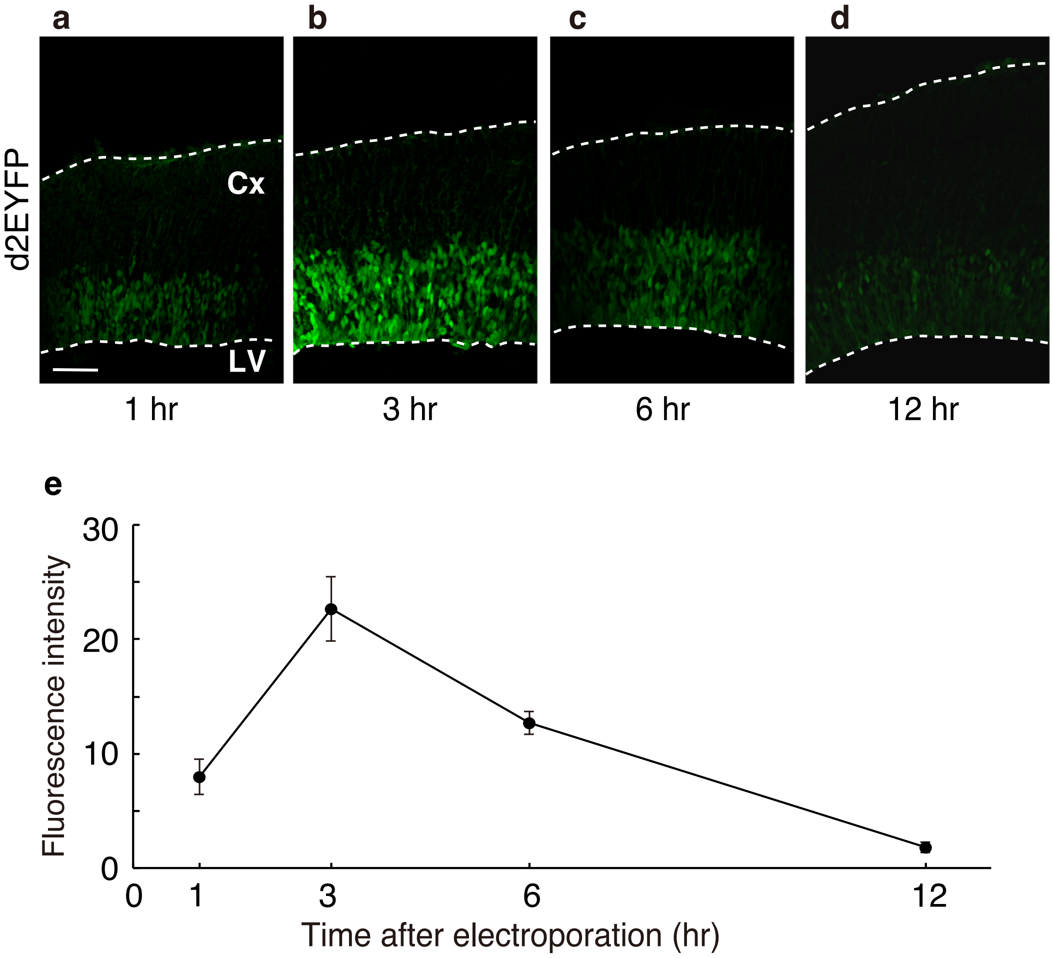

Supplementary Fig. S3. Transient d2EYFP expression after mRNA electroporation. The *d2EYFP* mRNA containing m1Ψ was transfected as described in Fig. 1. **a-d,** Coronal sections of the Cx, one to 12 hours after electroporation. Scale bar, 50 µm. **e,** Fluorescence intensity of d2EYFP-positive areas, one to 12 hours after electroporation. Data are presented as mean ± s.e.m. $n = 5$ electroporated brains.

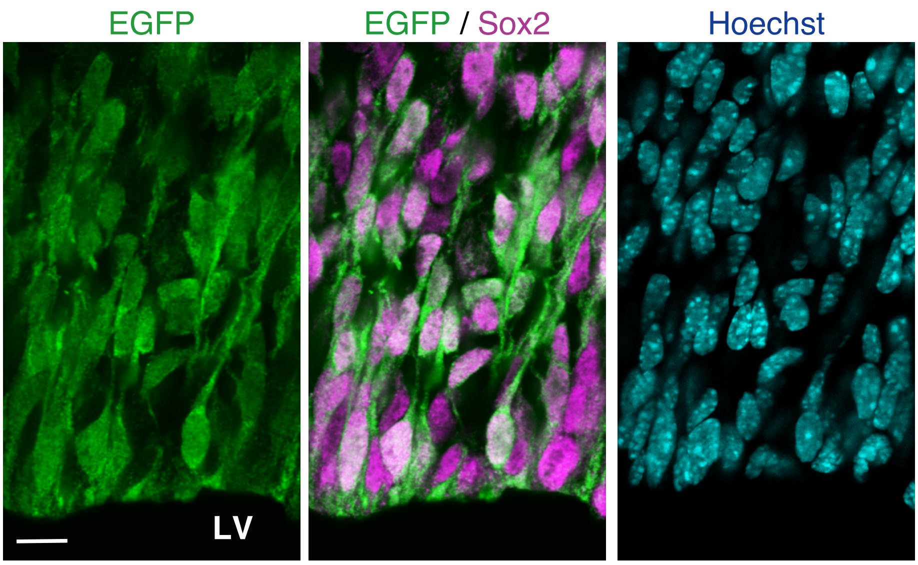

Supplementary Fig. S4. EGFP-positive cells express the neural stem cell marker Sox2. High magnification images of the ventricular zone of the Cx, one hour after *EGFP* mRNA electroporation performed as described in Fig. 1. The coronal section was immunostained with anti-EGFP (green) and anti-Sox2 (magenta) antibodies. Hoechst staining (blue) indicates nuclei. Scale bar, 20 µm. $n = 3$ electroporated brains.
